# Role of Advanced MRI-Based Perfusion and Diffusion Imaging in Predicting Recovery After the Surgical Management of Cervical Myelopathy: A Retrospective Study

**DOI:** 10.7759/cureus.104234

**Published:** 2026-02-25

**Authors:** Adeel Ur Rehman, Irfan Adil, Aashita Singh, Syed Hassan Raza Bokhari, Gyanendra K C, Kester O Maduadi, Zahra Ali, Abdul Waheed Bahir

**Affiliations:** 1 Neurological Surgery, Punjab Institute of Neurosciences, Lahore, PAK; 2 Neurosurgery, Bolan Medical College, Quetta, PAK; 3 Orthopaedics, Orthonova Hospital, Jalandhar, IND; 4 Trauma and Orthopaedics, East Kent Hospitals University NHS Foundation Trust, Kent, GBR; 5 Radiology, KIST Medical College and Teaching Hospital, Lalitpur, NPL; 6 Radiodiagnosis, Vision XRAY Group, Sydney, AUS; 7 Obstetrics and Gynaecology, Sir Ganga Ram Hospital, Lahore, PAK; 8 Orthopaedic Surgery, Yan'an Hospital Affiliated to Kunming Medical University, Kunming, CHN

**Keywords:** cancer patients, cervical myelopathy, fractional anisotropy, postoperative recovery, prognostic biomarkers

## Abstract

Background: Cervical myelopathy is a common cause of non-traumatic spinal cord dysfunction, where both structural compression and microvascular compromise contribute to neurological deficits.

Objective: This study aimed to evaluate the prognostic value of advanced MRI-based perfusion and diffusion parameters in predicting neurological recovery following surgical decompression in patients with cervical myelopathy.

Methodology: This retrospective observational study was conducted at the Department of Neurosurgery, Punjab Institute of Neurosciences, Lahore, Pakistan, from September 2024 to September 2025 and included 105 patients with clinically and radiologically confirmed cervical myelopathy who underwent decompressive surgery. Preoperative MRI included diffusion tensor imaging (DTI)-derived parameters fractional anisotropy (FA), apparent diffusion coefficient (ADC), and intravoxel incoherent motion (IVIM) perfusion metrics true diffusion coefficient (D), pseudo-diffusion coefficient (D*), and perfusion fraction (f).

Results: The mean preoperative and postoperative Modified Japanese Orthopaedic Association (mJOA) scores were 10.4±2.3 and 14.9±1.8, respectively (p<0.001). FA showed a strong positive correlation with recovery rate (r=0.68; p<0.001), while ADC demonstrated a significant negative correlation (r=-0.59; p<0.001). Perfusion fraction also correlated positively with recovery (r=0.53; p<0.01). The receiver operating characteristic (ROC) analysis identified FA ≥0.41 and f ≥9.5% as optimal thresholds for predicting good recovery (area under the curve (AUC)=0.86 and 0.78, respectively). A combined FA+f model achieved the highest predictive accuracy (AUC=0.90).

Conclusion: It is concluded that advanced MRI-based diffusion and perfusion imaging provides a reliable, quantitative method for predicting postoperative recovery in cervical myelopathy. FA and perfusion fraction are the most effective predictors of neurological improvement, offering superior prognostic value compared to conventional MRI.

## Introduction

Cervical myelopathy, particularly degenerative cervical myelopathy (DCM), is the leading cause of non-traumatic spinal cord injuries worldwide in the adult population [1]. It occurs as a result of chronic compression of the spinal cord due to age-related changes in the DCM pathway, which includes, but is not limited to, intervertebral disc herniation, formation of an osteophyte, ossification of the posterior longitudinal ligament, and/or hypertrophy of the ligamentum flavum. These changes progressively compress the spinal canal, which results in the compression and ischemia of the spinal cord [2]. The resultant pathophysiological cascade consists of demyelination of the spinal cord, axonal degeneration, and neuronal loss due to microvascular compromise, which manifests in varying motor dysfunction and sensory disturbances [3]. The goal of surgical decompression in symptomatic patients is to stop the disease process and/or achieve a neurological plateau. The degree of functional recovery that remains is quite variable, even in patients with similar postoperative compression. This varies in patients, and several neurologically impaired patients exist, with no recovery despite successful decompression. This highlights the strong need for accurate, reliable, inexpensive, and non-invasive preoperative tools that can predict functional outcomes postoperatively and impact clinical decision-making.

MRI is a useful tool for both clinically diagnosing DCM and the neurological assessment to locate the level and extent of cord compression and degeneration [4]. Traditionally, edema, demyelination, or gliosis within the spinal cord, indicated by signal changes or hyperintensities on T2-weighted MRI scans, was believed to represent spinal cord injuries. Even with the presence of edema worsening postoperative prognosis in a majority of cases, a small percentage of patients with T2 signal changes or hyperintensities on their MRI scans exhibit little to no postoperative improvement [5]. Conversely, patients with cords appearing normal on MRI scans exhibit little to no postoperative improvement. While MRI scans illustrate anatomy reasonably well, they are mindfully inconclusive and tend to miss microstructural, hemodynamic changes that remain critical to the proper function and repair of the nervous system [6].

With advancements in MRI technology, particularly the shift toward functional imaging, it has become possible to examine the anatomy of the spinal cord in greater detail. Additionally, subtractive functional imaging techniques allow for the assessment of spinal cord activity and help address questions related to its hemodynamic function. This can be achieved using diffusion-weighted imaging (DWI), diffusion tensor imaging (DTI), and perfusion MRI sequences [7]. In diffusion imaging, the overall motion of water within the remaining spinal cord tissue is measured. In a healthy spinal cord, water diffusion occurs in a random and mixed or anisotropic fashion; this organized structure can become disutilized in cases of myelopathy, resulting in decreased fractional anisotropy (FA) and increased apparent diffusion coefficients (ADC) [8]. Various research has shown that diffusion parameters align with the degree of neurological damage and can identify early microstructural damage prior to conventional MRI manifestations [9]. Further, FA and ADC values analyzed before surgery have been associated with postoperative prognoses where decreased FA values are likely to forecast poor functional postoperative recovery and increased FA values are associated with significant recovery [10,11]. This evidence suggests that DTI values can function as a measurable indicator of the spinal cord condition and also of the injury that can or cannot be resolved with decompression [12].

The basic aim of the study is to evaluate the prognostic value of advanced MRI-based perfusion and diffusion parameters in predicting neurological recovery following surgical decompression in patients with cervical myelopathy.

## Materials and methods

This retrospective observational study was conducted at the Department of Neurosurgery, Punjab Institute of Neurosciences, Lahore, Pakistan, from September 2024 to September 2025. A total of 105 patients who met the inclusion criteria were included in the analysis. The study included adult patients between 18 and 75 years of age who had radiologically and clinically confirmed cervical myelopathy resulting from degenerative, compressive, or spondylotic etiologies. Only those who underwent surgical decompression, whether through anterior, posterior, or combined approaches, and possessed complete preoperative advanced MRI, including diffusion and perfusion sequences, were eligible. Additionally, patients were required to have fully documented neurological assessments both before surgery and at the six-month postoperative follow-up to ensure reliable comparison of outcomes. Patients were excluded if they had a history of previous cervical spine surgery or if their myelopathy resulted from non-degenerative conditions such as trauma, tumors, infections, or demyelinating disorders. Cases with incomplete MRI datasets or poor-quality images affected by motion artifacts were omitted, as were patients lacking postoperative follow-up or having incomplete clinical documentation, since these limitations would compromise the integrity of the data and analyses.

Data collection

The hospital's electronic medical record system was used to collect data. Samples of demographic details, details of the preoperative MRI, surgery details, and postoperative neurological outcomes were collected using a pre-tested data collection instrument. Imaging was conducted using a 3.0 Tesla MRI. Imaging was segregated into two categories: conventional and advanced. T1- and T2-weighted images were used to evaluate the anatomy of the canal and to determine the presence of cord compression and if there were signal changes. Advanced imaging was in the form of a diffusion and perfusion MRI and used the intravoxel incoherent motion (IVIM) technique. For DTI, the generation of diffusion maps was done using 32 gradient directions to extract the properties of FA, ADC, and mean diffusivity (MD). Metrics of perfusion imaging that were gotten from the IVIM were the true diffusion coefficient (D), pseudo-diffusion coefficient (D*), and perfusion fraction (f). Regions of interest (ROIs) were manually drawn at the point of maximal compression and at a nearby section that appeared normal. Clinical outcomes were kept from the two neuroradiologists reviewing the images, and average data was used for the study. In terms of the site, level, and nature of compression, all patients had undergone decompressive surgeries that were either anterior cervical discectomy and fusion (ACDF), posterior laminectomy, or laminoplasty.

Data analysis

Statistical analysis was performed using IBM SPSS Statistics for Windows, Version 26.0 (IBM Corp., Armonk, New York, United States). Continuous variables were summarized as mean±standard deviation, and categorical variables were presented as frequencies and percentages. Between-group comparisons were conducted using independent samples t-tests for continuous variables and appropriate categorical tests where required. Pearson's correlation coefficient was used to evaluate associations between imaging parameters and postoperative recovery rates. Multivariable logistic regression analysis was performed to identify independent predictors of poor neurological recovery. Variable selection followed a structured approach that prioritized clinically established predictors such as age, duration of symptoms, and baseline Modified Japanese Orthopaedic Association (mJOA) score, along with imaging parameters demonstrating statistical relevance in univariate analysis. Multicollinearity was assessed using variance inflation factor thresholds. Given that the smaller outcome group consisted of 44 poor recovery cases, the number of predictors included in the final model was restricted to four variables to maintain an approximate 10 events-per-variable ratio and reduce the risk of overfitting. Adjusted odds ratios with 95% confidence intervals were calculated, and a p-value of <0.05 was considered statistically significant.

## Results

Data were collected from 105 patients, and the mean age of the cohort was 54.6±9.8 years, with a predominance of males (68, 64.8%). The average symptom duration before surgery was 8.2±3.5 months. The most common presenting complaints were limb weakness (76, 72.4%), gait disturbance (62, 59%), neck pain (58, 55.2%), and sensory deficits (47, 44.8%). Hypertension and diabetes mellitus were the leading comorbidities, reported in 38.1% and 26.7% of patients, respectively. The level most frequently affected was C5-C6 (44, 41.9%), followed by C4-C5 (34, 32.4%). The mean spinal canal diameter at the narrowest segment was 8.6±1.7 mm, and T2 hyperintensity within the cord was detected in 82 (78.1%) of cases, indicating varying degrees of myelopathic change. Quantitative MRI parameters revealed the mean values of FA at 0.432±0.089, ADC at 1.23±0.21×10⁻³ mm²/s, true diffusion coefficient (D) at 1.05±0.18×10⁻³ mm²/s, pseudo-diffusion coefficient (D*) at 14.8±3.2×10⁻³ mm²/s, and perfusion fraction (f) at 9.7±2.1%, reflecting measurable microstructural and perfusion changes at the compressed spinal cord level (Table 1).

**Table 1:** Baseline demographic and clinical characteristics of the patients (n=105)

Variable	Mean±SD/n (%)
Age (years)	54.6±9.8
Gender	
Male	68 (64.8%)
Female	37 (35.2%)
Duration of symptoms (months)	8.2±3.5
Common symptoms	
Limb weakness	76 (72.4%)
Gait disturbance	62 (59%)
Neck pain	58 (55.2%)
Sensory loss	47 (44.8%)
Comorbidities	
Hypertension	40 (38.1%)
Diabetes mellitus	28 (26.7%)
Most affected level	
C5-C6	44 (41.9%)
C4-C5	34 (32.4%)
C3-C4 and others	27 (25.7%)
Spinal canal diameter (mm)	8.6±1.7
T2 hyperintensity on MRI	82 (78.1%)
Preoperative MRI diffusion and perfusion	
Fractional anisotropy (FA)	0.432±0.089
Apparent diffusion coefficient (ADC) (×10⁻³ mm²/s)	1.23±0.21
True diffusion coefficient (D) (×10⁻³ mm²/s)	1.05±0.18
Pseudo-diffusion coefficient (D*) (×10⁻³ mm²/s)	14.8±3.2
Perfusion fraction (f) (%)	9.7±2.1

Among all procedures, ACDF was the most frequently performed surgery (58, 55.2%), followed by posterior laminectomy (34, 32.4%) and laminoplasty (13, 12.4%). The mean preoperative mJOA score was 10.4±2.3, which significantly improved to 14.9±1.8 six months after decompression (p<0.001). The mean recovery rate calculated by Hirabayashi's formula was 56.8±17.9%. Based on recovery rate, 61 patients (58.1%) were categorized as having good recovery (≥50%), while 44 patients (41.9%) had poor recovery (<50%) (Table 2).

**Table 2:** Surgical approach and postoperative outcomes

Variable	n (%)/mean±SD
Surgical approach	
Anterior cervical discectomy and fusion (ACDF)	58 (55.2%)
Posterior laminectomy	34 (32.4%)
Laminoplasty	13 (12.4%)
Preoperative Modified Japanese Orthopaedic Association (mJOA) score	10.4±2.3
Postoperative Modified Japanese Orthopaedic Association (mJOA) score (6 months)	14.9±1.8
Mean recovery rate (%)	56.8±17.9
Outcome group	
Good recovery (≥50%)	61 (58.1%)
Poor recovery (<50%)	44 (41.9%)

FA demonstrated a strong positive correlation with recovery rate (r=0.68; p<0.001), indicating that higher preoperative FA values are associated with better postoperative outcomes. In contrast, ADC showed a significant negative correlation (r=-0.59; p<0.001), suggesting that higher diffusivity corresponds to more severe axonal injury and poorer prognosis. Perfusion fraction (f) exhibited a moderate positive correlation (r=0.53; p<0.01), highlighting the importance of preserved spinal cord microvascular perfusion in predicting functional improvement. The true diffusion coefficient (D) showed a weak but statistically significant correlation (r=0.31; p=0.04), while the pseudo-diffusion coefficient (D*) did not reach statistical significance (p=0.21) (Table 3).

**Table 3:** Correlation between MRI parameters and postoperative recovery

MRI parameter	Correlation coefficient (r)	P-value	t-value
Fractional anisotropy (FA)	0.68	<0.001	7.06
Apparent diffusion coefficient (ADC)	-0.59	<0.001	-5.56
True diffusion coefficient (D)	0.31	0.04	2.48
Pseudo-diffusion coefficient (D*)	0.12	0.21	0.92
Perfusion fraction (f)	0.53	<0.01	4.76

Patients who achieved good recovery were younger (52.8±9.2 years vs. 56.9±10.1 years; p=0.04) and had significantly shorter symptom duration (6.7±2.8 vs. 10.3±3.7 months; p<0.001). T2 hyperintensity was more prevalent in the poor recovery group (90.9%) compared to the good recovery group (68.9%) (p=0.01), indicating greater irreversible cord damage. The mean spinal canal diameter was also smaller in poor outcome patients (8.1±1.8 mm vs. 8.9±1.5 mm; p=0.03). Furthermore, preoperative and postoperative mJOA scores were significantly higher in patients with good recovery (11.2±2.1 and 15.8±1.4, respectively) compared to those with poor recovery (9.4±2.4 and 13.6±1.9; p<0.001) (Table 4).

**Table 4:** Comparison of baseline characteristics between good and poor recovery groups

Variable	Good recovery (n=61)	Poor recovery (n=44)	P-value	t-value
Age (years), mean±SD	52.8±9.2	56.9±10.1	0.04	-2.06
Gender (male/female)	40/21	28/16	0.87	-
Duration of symptoms (months)	6.7±2.8	10.3±3.7	<0.001	-5.07
Hypertension, n (%)	20 (32.8%)	20 (45.5%)	0.18	-
Diabetes mellitus, n (%)	13 (21.3%)	15 (34.1%)	0.16	-
T2 hyperintensity on MRI, n (%)	42 (68.9%)	40 (90.9%)	0.01	-
Spinal canal diameter (mm)	8.9±1.5	8.1±1.8	0.03	2.22
Preoperative Modified Japanese Orthopaedic Association (mJOA) score	11.2±2.1	9.4±2.4	<0.001	4.01
Postoperative Modified Japanese Orthopaedic Association (mJOA) score (6 months)	15.8±1.4	13.6±1.9	<0.001	6.61
Mean recovery rate (%)	67.2±11.3	41.7±9.2	<0.001	11.88

Fractional anisotropy (FA ≥0.41) emerged as the strongest independent predictor (OR 3.62; 95% CI 1.82-7.21; p<0.001). Lower ADC values (≤1.27×10⁻³ mm²/s) were also significantly associated with favorable outcomes (OR 2.91; p=0.002). Perfusion fraction ≥9.5% (OR 2.34; p=0.02) and shorter symptom duration ≤6 months (OR 2.85; p=0.004) independently predicted good recovery, while the absence of T2 hyperintensity showed a modest but significant association (OR 1.98; p=0.046). Age ≤55 years did not reach statistical significance (p=0.18) (Table 5).

**Table 5:** Multivariate logistic regression analysis of predictors of good postoperative recovery

Variable	Odds ratio (OR)	95% confidence interval (CI)	P-value
Fractional anisotropy (FA ≥0.41)	3.62	1.82-7.21	<0.001
Apparent diffusion coefficient (ADC ≤1.27×10⁻³ mm²/s)	2.91	1.48-5.74	0.002
Perfusion fraction (f ≥9.5%)	2.34	1.12-4.87	0.02
Duration of symptoms ≤6 months	2.85	1.37-5.91	0.004
Absence of T2 hyperintensity	1.98	1.01-3.87	0.046
Age ≤55 years	1.64	0.79-3.42	0.18
Constant	-	-	0.001

## Discussion

This research showed that advanced MRI diffusion and perfusion imaging techniques offer substantial prognostic insight regarding postoperative neurological recovery outcomes for patients who undergo surgical decompression for cervical myelopathy. When looking at all of the quantitative variables, FA and perfusion fraction (f) were the strongest correlates of postoperative functional recovery, while ADC showed an inverse relationship. This reaffirms the understanding that the microstructural integrity and microvascular perfusion of the spinal cord (as captured by diffusion and perfusion MRI techniques) are essential components that influence the extent of postoperative neurological recovery.

Previous studies that showed conventional MRI offered little utility to predict postoperative outcome corroborate the conclusions drawn here. Historically, T2-weighted hyperintensities were viewed as indicating cord injury, but their predictive value has consistently proved insufficient [12]. Several researchers have noted that the presence of T2 hyperintensities may reflect reversible or irreversible injury along a continuum that includes edema, demyelination, and myelomalacia pathologies that are indiscernible on conventional imaging. DTI addresses this disappointment, as it provides a means to both quantitatively and reproducibly evaluate the extent of axonal disorganization and white matter integrity. Postoperative improvement and greater ADC values that had an inverse and negative relationship on the surface may reflect greater axonal injury and gliosis [13,14]. These findings collectively suggest that lower FA values and higher ADC signify irreversible structural injury, with remaining anisotropy reflecting the chance of functional recovery after decompression.

The findings from perfusion imaging bolster this analytical approach even more. When spinal cords are under chronic compression, there is soft tissue deformation that damages the microvasculature, further compounding ischemic injuries [15]. In our study cohort, lower perfusion fraction values were significantly correlated with less favorable outcomes, suggesting that even less spinal cord blood flow may further hinder the dissipation of repairable tissue. This observation is consistent with previous studies that employed IVIM and arterial spin labeling and found that symptomatic cervical myelopathy patients had lower peroperative euvolumetric blood flow and benefited more from the procedures in terms of neurological function.

In our study, the moderate positive correlation of perfusion fraction with recovery rate (r=0.53; p<0.01) suggests that microvascular condition is an additional indicator that should be used with caution, along with the diffusion measurements [16]. Most notably, the combination of FA with perfusion fraction had the greatest predictive performance (area under the curve (AUC)=0.90) compared to either factor independently. This serves as a strong argument for using a multi-parameter imaging approach that combines microstructural and hemodynamic data to provide a richer characterization of the spinal cord. Other recent studies that employed multiple methods have argued that this approach is more likely to detect subtle changes and, therefore, take for usable changes in injured cords that may be recoverable. This observation is the most valuable outcome from this study [17,18].

In classical cervical myelopathy, prognosis depends mostly on subjective clinical grading and classical MRI morphology. However, sophisticated quantitative MRI techniques allow MRI-based risk stratification and adequate prognosis, which could also be used for deciding on surgical intervention and, further, patient counselling. Patients with preserved diffusion and perfusion should be stratified for early decompression. Patients with severely disrupted diffusion and perfusion should be treated with more rehabilitation strategies and appropriate goal setting. Also, for future clinical trials that focus on the neuroprotective or regenerative mechanisms, quantitative imaging biomarkers, as described, would be excellent target endpoints [19].

Despite its strengths, this study is not without limitations. Its retrospective design introduces inherent selection bias, and variations in surgical approach and postoperative rehabilitation could influence outcomes. Although imaging analysis was performed independently by two neuroradiologists to reduce observer bias, inter-scanner variability and technical limitations such as motion artifacts and low signal-to-noise ratio in spinal cord imaging remain challenges. Furthermore, perfusion imaging parameters such as pseudo-diffusion coefficient (D*) showed weaker associations, possibly reflecting the limitations of IVIM in capturing rapid microcirculatory changes within the narrow spinal cord. Prospective multicenter studies with standardized imaging protocols and longer follow-up durations are warranted to validate and generalize these findings.

## Conclusions

Advanced MRI-based perfusion and diffusion imaging provides a reliable and objective method for predicting neurological recovery after the surgical management of cervical myelopathy. The study demonstrates that FA and perfusion fraction (f) serve as the most significant prognostic indicators, showing a strong positive correlation with postoperative improvement, while higher ADC values are associated with poor recovery. These findings emphasize that microstructural integrity and microvascular perfusion within the spinal cord, quantified through advanced MRI sequences, play a decisive role in determining surgical outcomes.
